# Postmortem cardiac tissue maintains gene expression profile even after late harvesting

**DOI:** 10.1186/1471-2164-13-26

**Published:** 2012-01-17

**Authors:** Simone Gupta, Marc K Halushka, Gina M Hilton, Dan E Arking

**Affiliations:** 1 McKusick-Nathans Institute of Genetic Medicine, Johns Hopkins University School of Medicine, Baltimore, Maryland, USA; 2 Department of Pathology, Johns Hopkins University School of Medicine, Baltimore, Maryland, USA

## Abstract

**Background:**

Gene expression studies can be used to help identify disease-associated genes by comparing the levels of expressed transcripts between cases and controls, and to identify functional genetic variants (expression quantitative loci or eQTLs) by comparing expression levels between individuals with different genotypes. While many of these studies are performed in blood or lymphoblastoid cell lines due to tissue accessibility, the relevance of expression differences in tissues that are not the primary site of disease is unclear. Further, many eQTLs are tissue specific. Thus, there is a clear and compelling need to conduct gene expression studies in tissues that are specifically relevant to the disease of interest. One major technical concern about using autopsy-derived tissue is how representative it is of physiologic conditions, given the effect of postmortem interval on tissue degradation.

**Results:**

In this study, we monitored the gene expression of 13 tissue samples harvested from a rapid autopsy heart (non-failed heart) and 7 from a cardiac explant (failed heart) through 24 hours of autolysis. The 24 hour autopsy simulation was designed to reflect a typical autopsy scenario where a body may begin cooling to ambient temperature for ~12 hours, before transportation and storage in a refrigerated room in a morgue. In addition, we also simulated a scenario wherein the body was left at room temperature for up to 24 hours before being found. A small fraction (< 2.5%) of genes showed fluctuations in expression over the 24 hr period and largely belong to immune and signal response and energy metabolism-related processes. Global expression analysis suggests that RNA expression is reproducible over 24 hours of autolysis with 95% genes showing < 1.2 fold change. Comparing the rapid autopsy to the failed heart identified 480 differentially expressed genes, including several types of collagens, lumican (*LUM*), natriuretic peptide A (*NPPA*) and connective tissue growth factor (*CTGF*), which allows for the clear separation between failing and non-failing heart based on gene expression profiles.

**Conclusions:**

Our results demonstrate that RNA from autopsy-derived tissue, even up to 24 hours of autolysis, can be used to identify biologically relevant expression pattern differences, thus serving as a practical source for gene expression experiments.

## Background

Gene expression studies monitor the simultaneous transcription levels of genes, and thus bridge the gap between static genomic information and dynamic phenotypes. These studies are used to identify differentially expressed transcripts with respect to measured variables of interest, such as differing environments, treatments, phenotypes, or clinical outcomes, and for the identification of expression quantitative trait loci (eQTL), i.e. genomic regions whose genotype is correlated with the RNA expression in a panel of genetically diverse individuals [1]. Studies of gene expression abnormalities in various disorders often involve the use of postmortem tissue [2]. The factors affecting RNA stability or integrity in postmortem tissues have been most extensively studied in the neuropsychiatric area. Despite the success with identification of sets of differentially expressed genes specific to neuropsychiatric disorders, one can find continued concerns regarding quality and consistency of postmortem RNA analysis [3]. Prior studies in brain have indicated that specific agonal factors, including coma and hypoxia, markedly affect the integrity of RNA, whereas postmortem factors have relatively minor effects on RNA integrity [4]. Of the postmortem factors, pH is noted as a factor of importance for RNA quality in some publications [5].

Recent studies in human skeletal and heart tissues provide examples of variation in protein and enzyme activity, with examples of reduced NOS3 mRNA levels associated with higher postmortem interval (PMI) [6]. These alterations appear to occur more rapidly at higher temperatures, and storing samples at 4°C may delay some of the postmortem changes [7]. There has been a lingering debate over the importance of PMI on RNA degradation. Thus, in addition to variation in expression levels due to technical [8], environmental [9], demographic [10], and genetic factors [11], concerns arise as RNA integrity is affected by various pre- and postmortem events [12], which need to be addressed when quantitative analyses are performed.

These sources of variation raise a major technical concern about the utility of autopsy-derived tissue and how representative it is of physiologic conditions, due to the of the effect of PMI on tissue degradation. In this study we specifically test the utility of devitalized cardiac tissue for gene expression, obtained through both a cardiac explant (failed heart) and a rapid-autopsy program (non-failed heart). We systematically evaluate the variability of RNA message in devitalized tissue in a time dependent manner in an attempt to simulate general autopsy conditions.

Assessing the factors regulating RNA integrity is important to determine the reliability of gene expression patterns in cardiac tissue. Further, the existence of significant biological variability in expression levels of many genes between human hearts is widely recognized. Therefore, in this study we establish the extent of variation as a consequence of autolysis, and secondarily, generate a gene expression profile of a failing human heart.

## Methods

### Sample Collection

Cardiac tissue was obtained from an explanted failing heart via cardiac transplantation and from a "normal" heart through a metastatic cancer rapid-autopsy program. This study was approved by the IRB of The Johns Hopkins Hospital and all samples provided informed consent. Total RNA was extracted from the tissues using the RNeasy Fibrous Tissue Mini kit (Qiagen) according to the manufacture's protocol.

**Figure 1:**
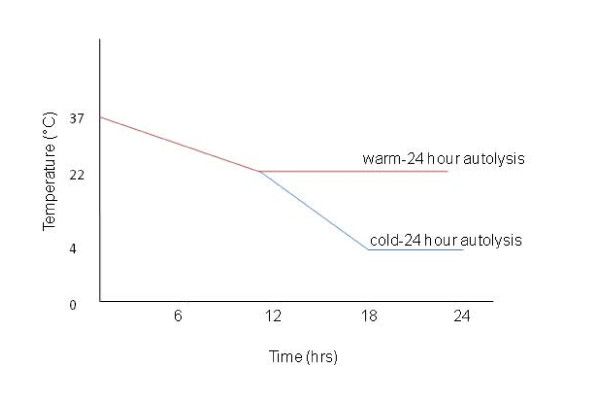
**Simulated autopsy conditions**. All heart samples were initially placed in a 37°C incubator that was then turned off and allowed to cool ~1°C/hr to reach room temperature ~12 hours later. Thereafter, to simulate cold and warm-24 hour autolysis tissue harvesting conditions, some hearts were cooled at 2-3°C/hr (to 4°C) (blue line) and others were left at room temperature (red line).

We obtained left and right ventricle tissue harvested under conditions that simulated general autopsy conditions. The baseline time-point (deemed the 0 hour time-point) for the explanted failing heart was harvested within 1 hour of explantation, while the autopsy heart was harvested 3-4 hours after the patient died. After rapidly collecting an initial 0 hour time-point to establish a baseline measure of gene expression, the hearts were placed in a 37°C incubator that was then turned off and allowed to cool at ~1°C/hr to reach room temperature ~12 hours later. After reaching ambient temperature, 6 heart samples were further cooled to 4°C (cold-24 hour autolysis) and 9 were left at room temperature (warm-24 hour autolysis). These two conditions represent two alternate scenarios of tissue harvesting. The first scenario simulates an autopsy where a body may begin cooling to ambient temperature for ~12 hours, before transportation and storage in a refrigerated room in a morgue which accelerates body cooling. The second we consider a "worst case" scenario where the body is not cooled. At each harvesting time point, tissue was flash frozen in liquid nitrogen or placed in RNAlater from the left and right ventricle free wall. Twenty tissue samples were harvested from each heart at time points 0, 6, 12, 18 (cold and warm) and 24 (cold and warm) hours (Figure 1). The RNA Integrity numbers (RIN) of all the 20 samples were obtained from the Agilent Bioanalyzer 2100 to assess the RNA quality of the samples [13]. Gene expression profiles were determined at time points 0, 6, 12, 18 and 24 hours in 13 tissue samples harvested from the autopsy heart. Profiles were obtained at time points 0, 6, 12, 18 hours in 7 tissue samples from the explant heart.

### Microarray Expression Analysis

RNA from the 20 samples, along with a HeLa control run in triplicate, were run on the Affymetrix Exon arrays according to the manufacturer's protocol. Array data for this study has been deposited in the National Center for Biotechnology Information's Gene Expression Omnibus (GEO) with accession number GSE32519. Exon arrays have 1.4 million probesets that map to over a million exon clusters. These feature probesets target each transcript throughout, rather than simply at the 3' end, allowing for robust identification of differentially expressed genes [14]. We analyzed expression data from ~200,000 core probesets that are supported by putative mRNAs from the RefSeq database. Signal processing was performed after quantile normalization and median polish summarization of the PM (Perfect Match) probes by using the Affymetrix power tools and the Robust Multi-Array Analysis (RMA) algorithm [15]. Probes with low p values (p < 0.05) have signals that are distinct from background and are considered to be detected above background (DABG). After filtering to retain probes flagged as present (DABG < 0.05) in at least one-fourth of the samples, 162,145 probes remained, which mapped to ~15,000 transcripts. Exon-based and intron-based probesets from putative housekeeping genes serve as positive and negative control sets, respectively. These signals are used to produce an "area under the curve" (AUC) value in a receiver-operating characteristic (ROC) curve as a measure of separation between the positive and negative controls, which is indicative of the quality of hybridization [16]

GNF Expression Atlas 2 [17] allows for the classification of genes depending on their expression in the 79 human organs and tissues covered by the Atlas. Approximately, 8,400 genes expressed in the heart in the GNF Expression Atlas were downloaded via the Biomart tool.

### Statistical Analysis

All statistical analysis was performed using R (version 2.9.2). Principal component analysis was performed using the function "prcomp" in the "stats" package of R. Two-class Significance Analysis of Microarrays (SAM) [18] was used to identify genes that were differentially expressed, which is implemented as the "samr" package in R. SAM calculates the test statistic for relative difference in gene expression based on permutation analysis of expression data and calculates a false discovery rate, which gives the proportion of significant genes most likely identified by chance. We used the two class unpaired test statistic in SAM. The two class unpaired test is similar to a between subjects t-tests wherein mean expressions in two groups are determined significant based on the calculated statistic, *d*, from the original and permuted data for each gene. In this study a false discovery rate (FDR; *q *< 5%) was used to determine the differential genes between the two classes.

### Functional Analysis

Genes were functionally annotated and classified by using the functional annotation tool of database for annotation, visualization, and integrated discovery (DAVID) [19]. The annotations were selected from Gene Ontology's "biological process". The annotations have been selected based on medium stringency (default) setting, with > 2-fold enrichment and Benjamini-Hochberg p-value < 0.05. The fold enrichment for a particular functional category describes the ratio between the number of genes in the gene list belonging to that category and the total number of genes in the gene list, which has at least one functional annotation. This ratio is then compared to the ratio between the total number of genes in that category and the total number of genes in the human genome with at least one functional annotation. It has been observed that it can be difficult to come up with a clear picture of gene function given all the Gene Ontology (GO) terms annotating the genes. Therefore, all the significantly enriched GO categories were assigned their corresponding parent GO Slim annotation. The GO Slims are reduced versions of GO containing high-level, broader parent terms in the whole GO [20] (http://www.geneontology.org/GO.slims.shtml). This is feasible with GO because of the hierarchical parent and child relationships recorded between the terms. Note that we only use the GO Slim terms of biological process.

## Results and Discussion

**Figure 2:**
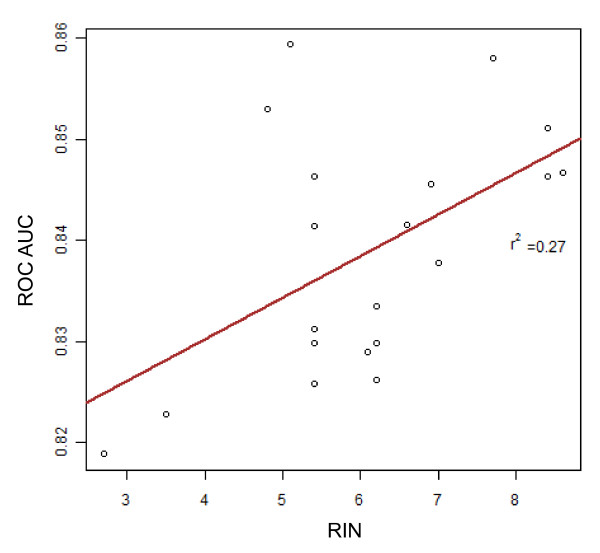
**RNA quality and hybridization performance**. The RIN number, a measure of RNA quality, ranged from 2.7-8.6 in the heart samples. The ROC AUC, a hybridization quality metric, ranged from 0.80 to 0.90, which is within the parameters suggested by Affymetrix. All samples showed robust hybridization irrespective of their RIN values (r^2 ^= 0.27).

**Figure 3:**
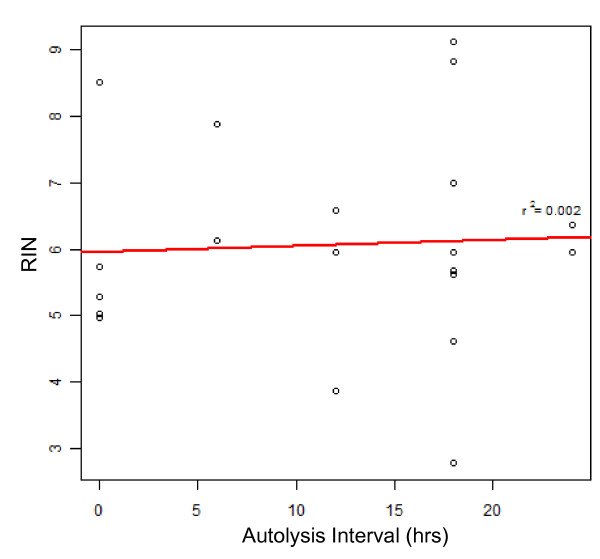
**Autolysis interval and RNA quality**. The autolysis interval, which ranged from 0 to 24 hours, did not correlate with RNA integrity, measured as RIN numbers (r^2 ^= 0.002).

We obtained the expression profiles of 7 cardiac tissue samples from the cardiac transplant operation (explant/failed heart) and 13 from a rapid-autopsy program (autopsy/non-failed heart) harvested under the "cold-" and "warm-24 hour autolysis" conditions (Figure 1). The left and right ventricular tissues showed highly correlated gene expression in both the heart samples (Pearson's coefficient, r > 0.94), and we therefore analyzed the heart regions together. To assess the expression array performance for each sample, we used the ROC AUC summarization score, which is a measure of overall sensitivity and specificity of the expression profile. The AUC scores for the 20 gene expression profiles harvested at increasing autolysis delay all ranged from 0.80 to 0.90, which is within the parameters suggested by Affymetrix [16]. While RIN values are typically used to predict sample performance, we only observed a weak correlation between RIN values and ROC AUC (r^2 ^= 0.27, Figure 2), and note that even samples with RIN values as low as 2.7 displayed robust hybridization. The means of the RIN values in the "warm" and "cold" 24 hour autolysis samples were 5.1 (SD = 1.5) and 6.9 (SD = 1.5), respectively, though this difference was not statistically significant (P = 0.11) (Additional File 1, Figure S1). We further observed that the RIN values were independent of the autolysis time (r^2 ^= 0.002, Figure 3). While this observation is based on a small dataset and is at odds with many, but not all, reports of RIN quality correlating with post mortem interval [21], we note that in our analyses, RIN was not a strong predictor of array performance.

### Autolysis Stability of Gene Expression

**Figure 4:**
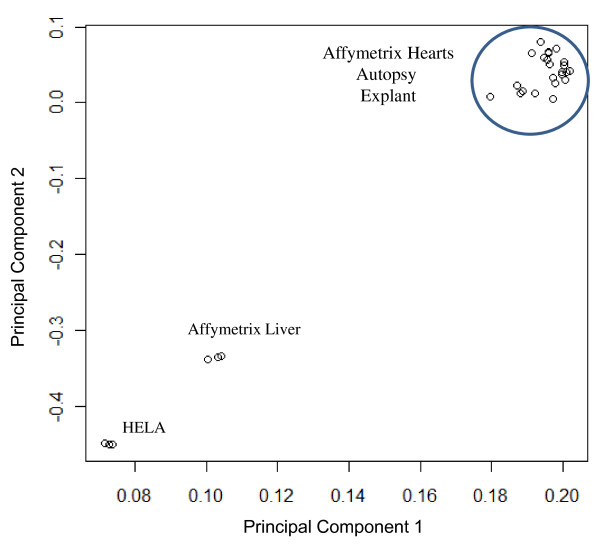
**Principal component analysis (PCA) of heart and non-heart gene expression intensities**. A scatterplot of the PC1 and PC2 from the PCA analysis of gene intensities of ~15,000 transcripts from the 20 autopsy and explant heart samples, along with reference triplicate HeLa controls and triplicate heart and liver data (obtained from Affymetrix), clearly separates the samples into heart and non-heart spaces.

**Figure 5:**
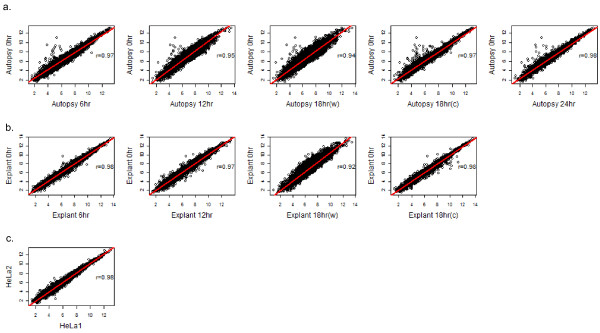
**Correlation between baseline and autolyzed gene expression**. **a **and **b**. Scatterplots of expression intensity later time-points (x-axis) and the 0 hour time-point (y-axis) for (a) autopsy and (b) explant heart tissues. Red lines represent the linear regression line indicating relationship between expression profiles. Pearson's correlation (r), between the baseline and later timepoints, range from 0.92-0.98. **c**. Scatterplot of expression intensities between two technical replicates (HeLa Controls) with Pearson's correlation, (r) of 0.98.

As an additional general test of expression array performance with autolysed tissue, we used principal component analysis (PCA) of gene intensities to cluster the 20 autopsy and explant heart samples, along with Hela controls run in triplicate, as well as reference liver and heart data obtained from Affymetrix. Irrespective of the time and temperature of tissue harvest, the expression profiles of the two hearts could clearly be differentiated from non-heart tissues, as observed by the PCA analysis of ~15,000 gene intensities (Figure 4). In Figure 5, we demonstrate that there is a strong correlation between the baseline explant and autopsy hearts compared to their later timepoints (Pearson's correlation ranges from 0.92-0.98). As a comparison, the Pearson's correlation(r), between any two HeLa controls (technical replicates) was 0.98. Additionally, the Pearson's coefficient for HeLa controls and reference liver with respect to a high RIN heart (RIN = 8.6) was 0.65 and 0.66, respectively.

**Figure 6:**
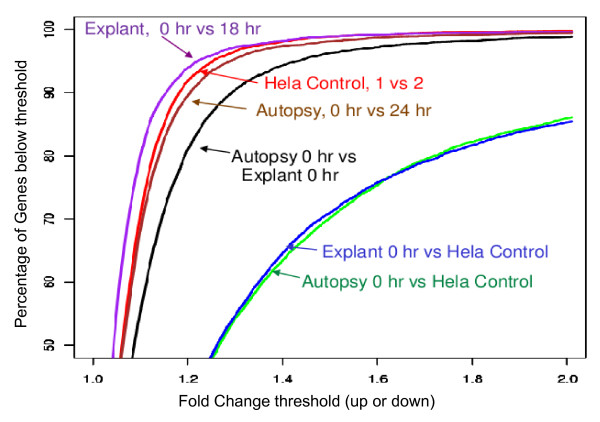
**Concordance in expression profiles in heart tissues**. Pair-wise comparisons of all cardiac expressed genes (~8,400 based on the GNF anatomical system data: heart), expressed as the fold change between time-points. To establish a baseline level of expected concordance for technical replicates, we compared the expression profile of two HeLa controls, and observed 94.9% of genes with ≤1.25 fold change (red line). Comparing the baseline explant and autopsy to later timepoints, we saw no difference from technical replicates (95.9% and 93.4% ≤1.25 fold change, respectively) (purple and brown lines). However, comparing the autopsy to the explant, we saw a measurable difference (86.8% ≤1.25 fold change) (black line), and comparing either heart sample to the HeLa controls demonstrated a striking difference (48% ≤1.25 fold change) (green and blue lines).

The concordance in expression profiles in autopsy tissue was also investigated by pair-wise comparisons of all cardiac expressed genes, expressed as the fold change between time-points, (Figure 6). Specifically, 8,400 genes expressed in the cardiac tissue were retrieved from GNF Expression Atlas 2, which summarizes the expression patterns of human, mouse and rat genes in several selected tissues using whole-genome microarray experiments. To establish a baseline level of expected concordance for technical replicates, we compared expression profile of two HeLa controls, and observed 94.9% of genes with ≤1.25 fold change in expression (red line, Figure 6). The baseline explant and autopsy and their later timepoints, showed similar concordance in expression and we saw no difference from technical replicates (95.9% and 93.4% genes with ≤1.25 fold change, respectively) (purple and brown lines, Figure 6). We observed a difference between the autopsy and explant expression profiles, with only 86.8% genes with ≤1.25 fold change (black line, Figure 6). Comparing, either heart sample to the HeLa controls demonstrated a striking difference in expression, with only 48% with ≤1.25 fold change (green and blue lines, Figure 6). Thus, global gene expression analysis suggests that RNA expression is reproducible over 24 hours of autolysis and we could distinguish between heart and non-heart samples.

### Autolysis Fluctuations of Gene Expression

After, establishing that global expression is largely unaffected by autolysis interval, we looked at the small fraction of genes that were sensitive to autolysis conditions. We selected transcripts with > 2 fold-change (2-FC) between the baseline and later timepoints from each heart to evaluate the change in gene expression during the 24 hours of autolysis in a time dependent manner. For this analysis, we focused on the "cold-24 h autolysis", as this represents a typical autopsy scenario. Only 2.25% (345/15320) of transcripts from the autopsy hearts had a > 2-FC when we compared the transcript levels from the 4 later time-points (6, 12, 18, 24) to the baseline levels. We queried these genes in the DAVID annotation database to find significantly overrepresented GO terms. Table 1, provides the functional categories overrepresented with a > 2-fold enrichment, Benjamini-Hochberg p-value < 0.05 and the broader parent GO Slim term. There was significant overrepresentation of GO terms which associated with two GO Slim categories 1) "cellular metabolism" (76/354) and 2) "response to stimulus" (42/354).

**Table 1:** Annotations overrepresented in 2.25% genes fluctuating in the autopsy (non-failed) heart

Term^a^	GO Gene Count	GO Slim	GO Slim Gene Count
response to external stimulus	42		
response to wounding	27	response to stimulus	42
inflammatory response	19		

lipid metabolic process	41		
organic acid metabolic process	33		
carboxylic acid metabolic process	33		
oxoacid metabolic process	33		
cellular lipid metabolic process	30		
fatty acid metabolic process	16		
oxidation reduction	31		
alcohol metabolic process	22	cellular metabolism	76
energy derivation by oxidation of organic compounds	12		
carbohydrate biosynthetic process	10		
generation of precursor metabolites and energy	18		
monocarboxylic acid metabolic process	25		
cellular ketone metabolic process	33		

**^a^**All functional annotations are significantly overrepresented with > 2-Fold Enrichment and Benjamini-Hochberg p-value < 0.05

We performed a similar experiment with the explanted heart, to see which categories of genes fluctuate in response to autolysis under the same conditions. One percent (154/15320) of transcripts from the explanted hearts had a > 2-FC, when we compared the fold changes in the transcripts from the 3 later time-points (6, 12, 18) to the baseline. Significantly overrepresented functional categories from the DAVID annotation database and the associated broader GO Slim parent terms are shown in Table 2. In the explant heart the significant enrichment of GO terms associated with the "response to stimulus" category was observed annotating 44/154 genes. Three other significant functional categories were mapped to "circulatory system process" (8/154), "development" (15/154) and "cell differentiation" (27/154). Thus, the broad parent GO Slim term "response to stimulus" was significantly overrepresented in both the autopsy and explant hearts, including 21 genes in common (Additional File 2, Table S1), in this analysis of variation in gene expression during 24 hours of autolysis.

**Table 2:** Annotations overrepresented in 1% genes fluctuating in the explant (failed) heart

Term^a^	GO Gene Count	GO Slim	GO Slim Gene Count
circulatory system process	8	circulatory system process	8

response to external stimulus	23		
regulation of defense response	9		
regulation of response to stimulus	15		
response to chemical stimulus	26		
regulation of inflammatory response	7		
response to cytokine stimulus	7		
response to corticosteroid stimulus	7		
response to organic substance	18		
response to extracellular stimulus	10		
response to mechanical stimulus	6		
response to endogenous stimulus	13		
response to stress	29		
response to inorganic substance	9	response to stimulus	44
regulation of response to external stimulus	8		
response to drug	9		
response to glucocorticoid stimulus	6		
response to lipopolysaccharide	6		
regulation of response to stress	10		
response to molecule of bacterial origin	6		
response to hormone stimulus	11		
response to steroid hormone stimulus	8		
response to nutrient levels	8		
response to bacterium	8		
transforming growth factor beta receptor signaling pathway	5		

skeletal system development	15	development cell	15

cell differentiation	27	differentiation	27

**^a^**All functional annotations are significantly overrepresented with > 2-Fold Enrichment and Benjamini-Hochberg p-value < 0.05

### Gene Expression Profile for a Failing Heart

**Figure 7:**
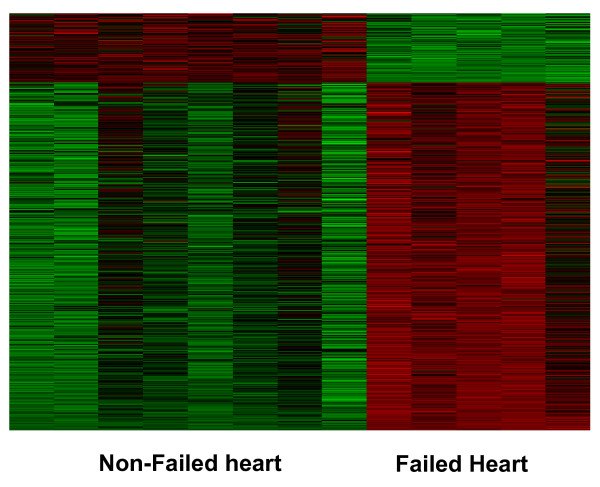
**Differential gene expression**. Heatmap of transcripts with > 2 fold change in expression between the failing and non-failing heart; 374 genes are up-regulated and 108 down-regulated. (red = up-regulated, and green = down-regulated)

Having established that the global expression from the individual cardiac tissues is reproducible during 24 hours of autolysis, we next compared the expression profile of the ventricular cardiac tissue from the explant (failing heart) and the autopsy (non-failing heart) by using SAM's two class unpaired test statistic. We focused on the "cold-24 hour autolysis" heart samples, as representative of real-world autopsy conditions. Gene expression profiles from 8 autopsy and 5 explant heart samples were analyzed with the SAM technique, which identified ~480 differentially expressed (DE) transcripts at > 2-FC and q-values < 0.5. The heatmap in Figure 7 demonstrates ~480 transcripts with > 2-FC expression between the two groups with 374 up-regulated and 108 down-regulated transcripts in the explant/failed heart. The list of ~480 genes differentially up- and down-regulated between the explant and autopsy hearts are given in the Additional File 3, Table S2. The results were unchanged after exclusion of the samples harvested at the baseline 0 hour time-point.

Clinical evidence clearly demonstrates the dynamic nature of the components of the extracellular matrix in response to mechanical unloading of the failing heart [22,23] and a quantitative increase in collagen subtypes [24]. We identified ECM proteins collagen I alpha 1 (*COL1A1*), collagen III alpha 1 (*COL3A1*) and fibronectin (*FN1*) to be upregulated > 2-FC in the failing heart. Importantly, we identified natriuretic peptide A (*NPPA*) which was 11-fold higher in the failing heart. *NPPA *is part of a conserved adaptive change in molecular phenotype in response to heart failure and serves as both a diagnostic and potential therapeutic marker [25,26]. Recently, higher than normal levels of osteoglycin (*OGN*, 12-fold increase in the failing heart) were associated with the heart becoming enlarged in rats and mice and in humans [27]. Periostin, (*POSTN*, 8-fold increase in the failing heart) has also been implicated in cardiac remodeling and therefore heart failure [28]. Additionally, the expression of connective tissue growth factor (*CTGF*, 4 fold change), transforming growth factor-beta (*TGFB2*, 3.18 fold change), brain natriuretic peptide precursor (*NPPB*, 2.29 fold change), Mu-crystallin (*CRYM, 2.8 *fold change*) *and clusterin (*CLU*, 2.5 fold change), were all elevated in the failing heart, consistent with previous reports [29-34]. The expression of Alpha-1-antichymotrypsin (*SERPINA3*), a known protease inhibitor, responsible for degradation or disassembly of myocardial proteins, was down regulated in the failing heart when compared to the non-failing heart (8 fold decrease), which is consistent with the report by Yang et al [35].

**Figure 8:**
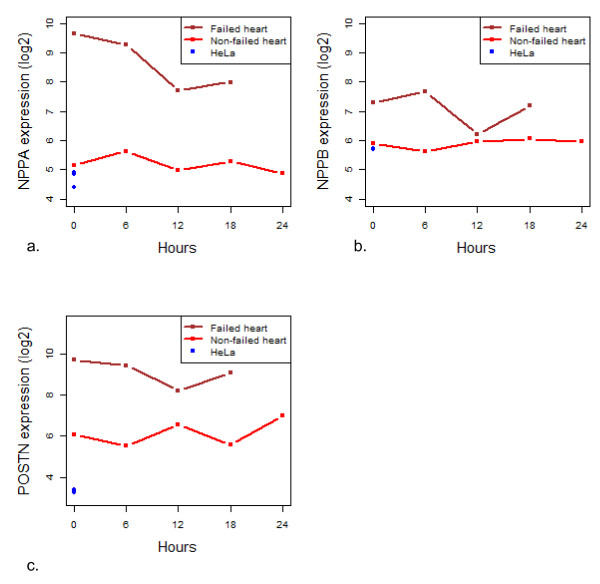
**Differentially expressed and fluctuating genes**. Expression profile of (a) *NPPA*, (b) *NPPB *and (c) *POSTN *genes in the failing and non-failing hearts. The genes are differentially expressed and show fluctuation in expression in the failed heart.

We also queried with the DAVID database to look for enrichment of functions from GO biological processes represented by the DE genes. The significantly over-represented functional annotations (> 2-fold enrichment and Benjamini-Hochberg p-value < 0.05) and the associated GO Slim terms are listed in Tables 3 and 4. Seventy-nine of the 374 up-regulated genes in the failing heart were annotated into overlapping overrepresented GO functional categories, with the associated GO slim terms "cellular metabolism" and "response to stimulus" annotated for 29/374 and 23/374 genes, respectively (Table 3). Fifty-nine of the 108 down-regulated genes belonged to overlapping functionally overrepresented GO categories which related to parent GO Slim terms "response to stimulus" (49/108), "regulation of biological quality" (23/108), "cellular process" (9/108), and "transport" (5/108) (Table 4). Given that we observed an enrichment of genes involved in "response to stimulus" in the analysis of genes that fluctuate during autolysis, we investigated whether the ~480 DE genes between the failing and non-failing heart included genes identified as fluctuating during autolysis. We observed that 13% of the DE genes showed temporal fluctuation in the autopsy hearts, while 9% showed fluctuation in the explants, including important marker genes like *NPPA*, *NPPB *and *POSTN*. We explored how fluctuation of these genes during autolysis influenced the comparison of gene expression between failed and non-failed heart. In all three cases, these genes were clearly able to distinguish failed from non-failed heart at all time points, with the exception of *NPPB *at the 12 hr timepoint (Figure 8). Thus, despite > 2FC fluctuations in temporal expression in some important genes due to autolysis, it is still possible to clearly identify specific and relevant differentially expressed genes in autolyzed cardiac tissue.

**Table 3:** Annotations overrepresented in the up-regulated genes in the explant (failed) heart

Term^a^	GO Gene Count	GO Slim	GO Slim Gene Count
cellular ketone metabolic process	29	cellular metabolism	29

extracellular structure organization	15	extracellular structure organization and biogenesis	15

muscle contraction	12	muscle system process	12

regulation of cell adhesion	13	regulation of biological process	13

enzyme linked receptor protein signaling pathway	23	response to stimulus	23

**^a^**All functional annotations are significantly overrepresented with > 2-Fold Enrichment and Benjamini-Hochberg p-value < 0.05

**Table 4:** Annotations overrepresented in the down-regulated genes in the explant (failed) heart

Term^a^	GO Gene Count	GO Slim	GO Slim Gene Count
cellular ion homeostasis	9	cellular process	9

regulation of biological quality	27	regulation of biological quality	27

defense response	22		
response to stimulus	48		
response to chemical stimulus	22		
immune system process	19		
response to stress	34		
immune response	14		
regulation of response to stimulus	12		
response to external stimulus	26		
response to bacterium	7		
response to wounding	20	response to stimulus	49
regulation of immune response	9		
regulation of defense response	7		
inflammatory response	16		
blood coagulation	6		
innate immune response	10		
positive regulation of defense	5		
response	9		
humoral immune response	6		
complement activation, classical pathway			

transition metal ion transport	5	transport	5

**^a^**All functional annotations are significantly overrepresented with > 2-Fold Enrichment and Benjamini-Hochberg p-value < 0.05

## Conclusion

In this study we investigated the gene expression of cardiac tissues in a simulated autopsy experiment. We allowed the heart tissue to autolyze for 24 hours, either at ambient temperature or shifted to 4°C after 12 hours. We chose a limit of 24 hours as we have shown previously that protein expression remains intact for 24 hours by immunohistochemistry, but degradation is noticeable at 48 hours [36]. Thus our findings are applicable to a 24 hour time course and we cannot speculate on late harvesting. We note that, in our experience, 24 hours is a reasonable amount of time to conduct a standard autopsy and is well within the time achievable by organ procurement organizations (OPOs) [37]. The vast majority of genes expressed are stable despite extended autolysis and reduced RNA integrity, indicating that multiple-probe based exon arrays are suited to reliably determine gene expression profiles. We found that most molecular processes are not subject to variation due to tissue procurement time and/or tissue storage temperature. Global expression analysis suggests that RNA expression is reproducible over 24 hours of autolysis with 95% genes showing < 1.2 fold change. As a proof of principle, we used SAM analysis to identify a list of 480 differentially expressed genes, including several types of collagens, lumican (*LUM*), natriuretic peptide A (*NPPA*) and connective tissue growth factor (*CTGF*), which allow for the clear separation between the failing and non-failing heart expression profiles - irrespective of autolysis time.

Our study is consistent with those that suggest that autolysis has relatively minor effects on RNA integrity. Only a small fraction (< 2.5%) of transcripts fluctuate over the 24 hours of autolysis, enriched in functional categories (energy metabolism, immune and signal responses) that are known to be the most adaptable and responsive biological processes and often highly variable between biological specimens [38]. Thus, our results demonstrate that RNA from autopsy-derived tissue, even up to 24 hours of autolysis, can be used to identify biologically relevant expression pattern differences, serving as a practical source for gene expression and eQTL experiments.

## Authors' contributions

SG carried out the analysis and drafted the manuscript. MKH obtained samples, contributed to the design of the experiment and helped in the draft of the manuscript; GMH extracted the RNA and conducted the experiments. DEA supervised the project, contributed to the design and coordination and helped draft the manuscript. All authors contributed to manuscript preparation and all approved the final manuscript.

## Supplementary Material

Additional file 1 **Figure S1: RIN range in "warm- and "cold-24 hour autolysis" samples**. The range of the RIN values in the warm-24 autolysis samples was 2.7 - 8.6, while the range in the cold- 24 autolysis samples was 3.5 - 8.6. The means of the RIN values in the "warm" and "cold" 24 hour autolysis samples were 5.1 (SD 1.53) and 6.9 (SD 1.45), respectively, and this difference was not statistically significant (P = 0.11). Click here for file

Additional file 2 **Table S1: Postmortem fluctuating genes common in autopsy and explant hearts**. The gene symbol and description of the 21 genes, which showed variation in gene expression during 24 hours of autolysis in both the autopsy and explant hearts. Click here for file

Additional file 3 **Table S2: List of 374 upregulated (Table S2a)and 108 downregulated (Table S2b) genes in the explant/failed heart**. List of ~480 genes differentially up- and down-regulated between the explant and autopsy hearts. Among these differentially expressed genes, 374 genes were upregulated and 108 genes were downregulated in the explant/failed heart. Click here for file
