# Supplementary figures and images for: Postmortem cardiac tissue maintains gene expression profile even after late harvesting
Source: BMC Genomics. 2012 Jan 17;13:26. doi: 10.1186/1471-2164-13-26 (PMC3342086; doi:10.1186/1471-2164-13-26)

## Slide 1
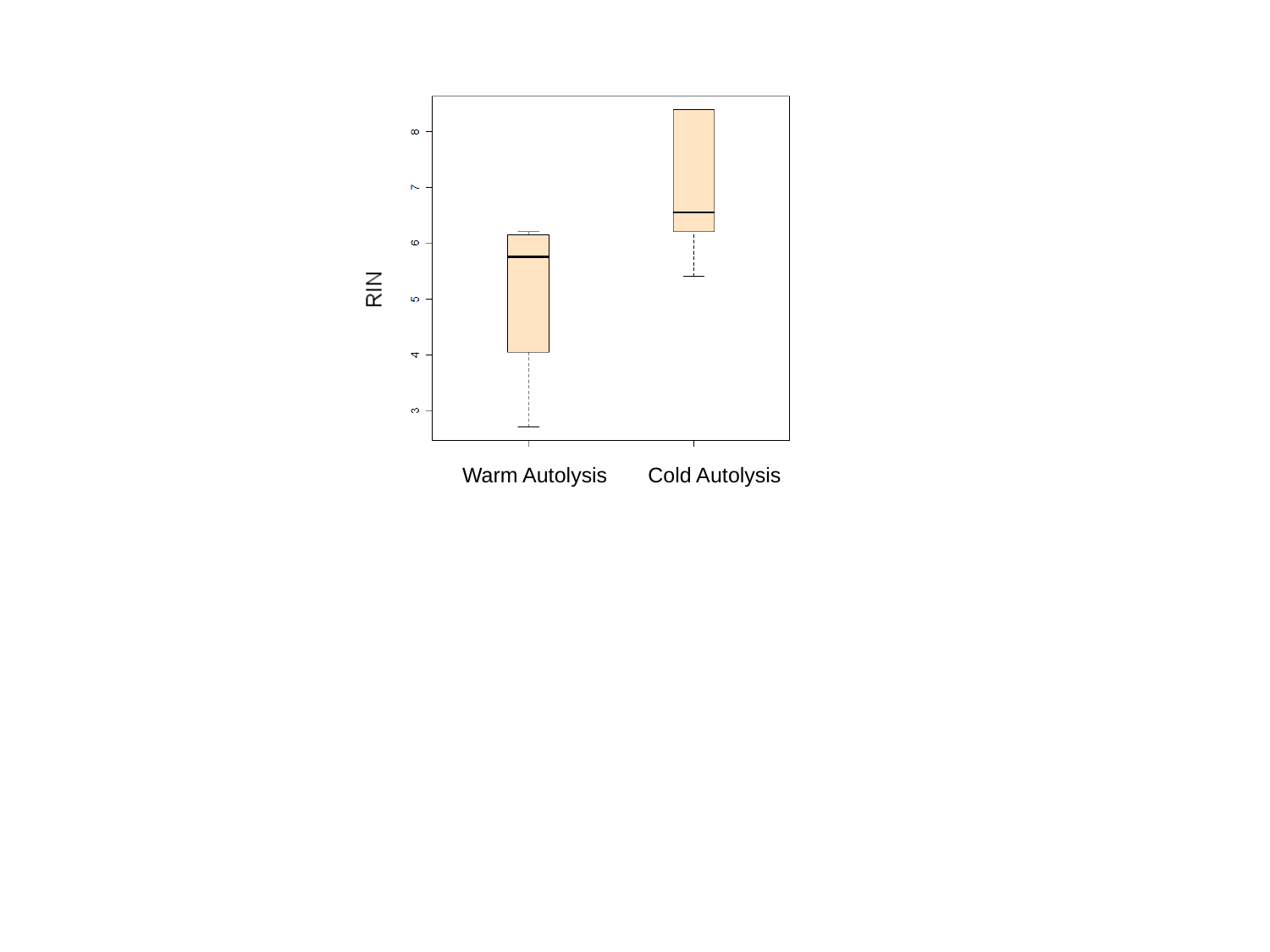

Warm Autolysis
Cold Autolysis

Supplement: Additional file 1 — Figure S1: RIN range in "warm- and "cold-24 hour autolysis" samples. The range of the RIN values in the warm-24 autolysis samples was 2.7 - 8.6, while the range in the cold- 24 autolysis samples was 3.5 - 8.6. The means of the RIN values in the "warm" and "cold" 24 hour autolysis samples were 5.1 (SD 1.53) and 6.9 (SD 1.45), respectively, and this difference was not statistically significant (P = 0.11). [file 1471-2164-13-26-S1.PPT]
