# Supplementary material for: Postmortem cardiac tissue maintains gene expression profile even after late harvesting
Source: BMC Genomics. 2012 Jan 17;13:26. doi: 10.1186/1471-2164-13-26 (PMC3342086; doi:10.1186/1471-2164-13-26)
Supplement: Additional file 2 — Table S1: Postmortem fluctuating genes common in autopsy and explant hearts. The gene symbol and description of the 21 genes, which showed variation in gene expression during 24 hours of autolysis in both the autopsy and explant hearts. [file 1471-2164-13-26-S2.DOC]

Table S1: Postmortem fluctuating genes common in autopsy and explant hearts

| **Gene Symbol** | **Description** |
| --- | --- |
| *CLIC6* | chloride intracellular channel 6 |
| *CHST9* | carbohydrate (N-acetylgalactosamine 4-0) sulfotransferase 9 |
| *C13orf30* | chromosome 13 open reading frame 30 |
| *MMRN1* | multimerin 1 |
| *SGPP2* | sphingosine-1-phosphate phosphatase 2 |
| *LOC284757* | hypothetical protein LOC284757 |
| *CRYM* | crystallin, mu |
| *CCL18* | chemokine (C-C motif) ligand 18 (pulmonary and activation-regulated) |
| *GKN2* | gastrokine 2 |
| *MAL2* | mal, T-cell differentiation protein 2 (gene/pseudogene) |
| *NDUFA8* | NADH dehydrogenase (ubiquinone) 1 alpha subcomplex, 8, 19kDa |
| *NDUFAF1* | NADH dehydrogenase (ubiquinone) 1 alpha subcomplex, assembly factor 1 |
| *CORIN* | corin, serine peptidase |
| *HOXC10* | homeobox C10 |
| *AMBN* | ameloblastin (enamel matrix protein) |
| *RBP4* | retinol binding protein 4, plasma |
| *CD69* | CD69 molecule |
| *IGK* | immunoglobulin kappa locus |
| *DPP4* | dipeptidyl-peptidase 4 |
| *CXCL14* | chemokine (C-X-C motif) ligand 14 |
| *ADIPOQ* | adiponectin, C1Q and collagen domain containing |
