# Supplementary material for: Postmortem cardiac tissue maintains gene expression profile even after late harvesting
Source: BMC Genomics. 2012 Jan 17;13:26. doi: 10.1186/1471-2164-13-26 (PMC3342086; doi:10.1186/1471-2164-13-26)
Supplement: Additional file 3 — Table S2: List of 374 upregulated (Table S2a)and 108 downregulated (Table S2b) genes in the explant/failed heart. List of ~480 genes differentially up- and down-regulated between the explant and autopsy hearts. Among these differentially expressed genes, 374 genes were upregulated and 108 genes were downregulated in the explant/failed heart. [file 1471-2164-13-26-S3.DOC]

**Table S2: List of 374 upregulated (Table S2a)and 108 downregulated (Table S2b) genes in the explant/failed heart.**

Table S2a: List of 374 Upregulated genes in the explant/failed heart.

| Gene ID | Fold Change | q-value(%) |
| --- | --- | --- |
| *ASPN* | 15.90 | 0 |
| *OGN* | 12.70 | 0 |
| *ACTA1* | 12.42 | 0 |
| *NPPA* | 11.56 | 0 |
| *ASB14* | 10.20 | 0 |
| *CXCL10* | 8.77 | 0 |
| *POSTN* | 8.74 | 0 |
| *SFRP4* | 8.41 | 0 |
| *OMD* | 7.77 | 0 |
| *GUCA1C* | 7.28 | 0 |
| *NPR3* | 6.98 | 0 |
| *ATRNL1* | 6.35 | 0 |
| *HAPLN1* | 5.96 | 0 |
| *FRZB* | 5.96 | 0 |
| *C4orf29* | 5.58 | 0 |
| *LEPREL1* | 5.58 | 0 |
| *STK17A* | 5.35 | 0 |
| *SFRP1* | 5.28 | 0 |
| *CASQ1* | 5.27 | 0 |
| *CCDC80* | 5.12 | 0 |
| *LUM* | 5.04 | 0 |
| *MLLT11* | 5.02 | 0 |
| *MXRA5* | 4.98 | 0 |
| *XIRP2* | 4.83 | 0 |
| *RASL11B* | 4.62 | 0 |
| *FAM180A* | 4.51 | 0 |
| *FBN2* | 4.51 | 0 |
| *IGJ* | 4.45 | 0 |
| *TNNT1* | 4.45 | 0 |
| *MME* | 4.42 | 0 |
| *EPHA7* | 4.36 | 0 |
| *EDNRA* | 4.25 | 0 |
| *FMOD* | 4.22 | 0 |
| *FILIP1L* | 4.13 | 0 |
| *OXCT1* | 4.11 | 0 |
| *LTBP2* | 4.02 | 0 |
| *DOK5* | 3.96 | 0 |
| *GRIA3* | 3.86 | 0 |
| *INPP4B* | 3.81 | 0 |
| *GATM* | 3.80 | 0 |
| *RERGL* | 3.79 | 0 |
| *COL14A1* | 3.76 | 0 |
| *IGK* | 3.73 | 0 |
| *DIRAS3* | 3.72 | 0 |
| *LRRC10* | 3.69 | 0 |
| *CTGF* | 3.69 | 0 |
| *GABRB1* | 3.69 | 0 |
| *VAT1L* | 3.67 | 0 |
| *DDAH1* | 3.59 | 0 |
| *NRK* | 3.55 | 0 |
| *DPT* | 3.52 | 0 |
| *PRKG2* | 3.48 | 0 |
| *PDP1* | 3.39 | 0 |
| *GBP4* | 3.36 | 0 |
| *SLC27A6* | 3.36 | 0 |
| *FGFBP2* | 3.33 | 0 |
| *IFI44L* | 3.33 | 0 |
| *CECR1* | 3.31 | 0 |
| *SMOC2* | 3.27 | 0 |
| *THBS4* | 3.23 | 0 |
| *SLC44A5* | 3.22 | 0 |
| *RGS4* | 3.21 | 0 |
| *TGFB2* | 3.19 | 0 |
| *ECM2* | 3.18 | 0 |
| *PCDH7* | 3.18 | 0 |
| *WBSCR17* | 3.17 | 0 |
| *TMEM74* | 3.14 | 0 |
| *LRRC39* | 3.14 | 0 |
| *VSNL1* | 3.14 | 0 |
| *FAP* | 3.13 | 0 |
| *NEB* | 3.10 | 0 |
| *EDIL3* | 3.08 | 0.033807449 |
| *GSTM5* | 3.06 | 0 |
| *SEMA6D* | 3.03 | 0 |
| *NR1D2* | 3.03 | 0 |
| *HCCS* | 3.01 | 0 |
| *LMOD3* | 3.00 | 0 |
| *KLHDC6* | 3.00 | 0 |
| *GPR34* | 2.99 | 0.015263661 |
| *CTSK* | 2.99 | 0 |
| *MARCH3* | 2.97 | 0 |
| *HPGDS* | 2.96 | 0 |
| *CPA3* | 2.95 | 0 |
| *LRRIQ3* | 2.92 | 0 |
| *LCLAT1* | 2.91 | 0 |
| *PPP1R3A* | 2.91 | 0 |
| *PRR16* | 2.91 | 0 |
| *CILP* | 2.90 | 0 |
| *FOSB* | 2.89 | 0.102880443 |
| *TLR7* | 2.89 | 0 |
| *CRYM* | 2.88 | 0 |
| *CYP2J2* | 2.87 | 0 |
| *ICK* | 2.85 | 0 |
| *IFI6* | 2.82 | 0 |
| *DIO2* | 2.82 | 0 |
| *C18orf19* | 2.81 | 0 |
| *IFIT1* | 2.81 | 0 |
| *ACOT11* | 2.80 | 0 |
| *MOXD1* | 2.79 | 0 |
| *NAP1L3* | 2.78 | 0 |
| *MYL7* | 2.78 | 0 |
| *ANO5* | 2.76 | 0 |
| *TFRC* | 2.76 | 0 |
| *CENPP* | 2.75 | 0 |
| *PIK3R1* | 2.75 | 0 |
| *C10orf71* | 2.74 | 0 |
| *CDCA7L* | 2.72 | 0 |
| *TNNT3* | 2.72 | 0 |
| *IGSF6* | 2.71 | 0 |
| *GABRE* | 2.71 | 0 |
| *TTLL7* | 2.69 | 0 |
| *ZDHHC2* | 2.69 | 0 |
| *PLEKHH2* | 2.68 | 0 |
| *HOMER1* | 2.67 | 0 |
| *BRP44L* | 2.67 | 0 |
| *CCDC93* | 2.67 | 0 |
| *CADPS* | 2.65 | 0 |
| *PI16* | 2.65 | 0 |
| *RBM24* | 2.64 | 0 |
| *FOS* | 2.63 | 0.301941203 |
| *ACTA2* | 2.63 | 0 |
| *POF1B* | 2.62 | 0.015263661 |
| *ST8SIA5* | 2.62 | 0 |
| *MLF1* | 2.59 | 0.015263661 |
| *CLU* | 2.59 | 0 |
| *CXCL9* | 2.58 | 0 |
| *GABRA4* | 2.58 | 0 |
| *OAS1* | 2.58 | 0 |
| *ASB2* | 2.56 | 0 |
| *CACNA2D1* | 2.56 | 0 |
| *TMEM144* | 2.56 | 0 |
| *CTNND2* | 2.55 | 0 |
| *COL3A1* | 2.54 | 0 |
| *SLC2A12* | 2.54 | 0 |
| *NT5C1A* | 2.54 | 0 |
| *HDAC9* | 2.53 | 0 |
| *IGSF10* | 2.53 | 0 |
| *GAB1* | 2.50 | 0 |
| *JAK2* | 2.49 | 0 |
| *LYPLAL1* | 2.49 | 0 |
| *ROR1* | 2.49 | 0 |
| *SCN5A* | 2.49 | 0 |
| *OSGEPL1* | 2.48 | 0 |
| *CHN2* | 2.48 | 0 |
| *HFE2* | 2.48 | 0 |
| *BDH1* | 2.47 | 0 |
| *SMAD6* | 2.47 | 0 |
| *BGN* | 2.46 | 0 |
| *C4orf18* | 2.46 | 0 |
| *RSAD2* | 2.45 | 0 |
| *IRX6* | 2.45 | 0 |
| *CLIC6* | 2.44 | 0.015263661 |
| *RRAGD* | 2.44 | 0 |
| *EHD3* | 2.44 | 0 |
| *FLJ34690* | 2.44 | 0 |
| *GNLY* | 2.44 | 0 |
| *KALRN* | 2.43 | 0 |
| *SLMAP* | 2.43 | 0 |
| *TRAK2* | 2.42 | 0 |
| *SPOCK1* | 2.41 | 0 |
| *SNX2* | 2.41 | 0 |
| *PXMP2* | 2.41 | 0 |
| *ACOT2* | 2.40 | 0 |
| *LIN9* | 2.39 | 0 |
| *SORCS1* | 2.39 | 0 |
| *RGN* | 2.38 | 0 |
| *NEK7* | 2.38 | 0 |
| *TYRP1* | 2.37 | 0 |
| *PAFAH2* | 2.37 | 0 |
| *IFIT2* | 2.37 | 0 |
| *GLT8D2* | 2.37 | 0 |
| *ACADM* | 2.36 | 0 |
| *GAS2* | 2.36 | 0 |
| *ZNF610* | 2.36 | 0 |
| *COL1A2* | 2.35 | 0 |
| *MATN2* | 2.35 | 0 |
| *DUSP19* | 2.35 | 0 |
| *SERTAD4* | 2.35 | 0 |
| *FASTKD1* | 2.34 | 0 |
| *SCRN3* | 2.34 | 0 |
| *ZNF277* | 2.34 | 0 |
| *MRPL34* | 2.34 | 0 |
| *C3orf23* | 2.34 | 0 |
| *PLK2* | 2.33 | 0 |
| *DPP4* | 2.32 | 0 |
| *GLDN* | 2.32 | 0 |
| *IFI44* | 2.32 | 0 |
| *NLGN4X* | 2.32 | 0 |
| *SLC38A3* | 2.32 | 0 |
| *NME5* | 2.31 | 0 |
| *LIAS* | 2.31 | 0 |
| *SUCLA2* | 2.31 | 0 |
| *ABCG2* | 2.31 | 0 |
| *FGF18* | 2.31 | 0 |
| *AGL* | 2.31 | 0 |
| *TSPAN12* | 2.30 | 0 |
| *CRIP3* | 2.30 | 0 |
| *RAP1A* | 2.30 | 0 |
| *NPPB* | 2.29 | 0 |
| *PRRX1* | 2.29 | 0 |
| *IRX3* | 2.29 | 0 |
| *OXNAD1* | 2.29 | 0 |
| *PRKAA2* | 2.29 | 0 |
| *ZNF382* | 2.28 | 0 |
| *RXRG* | 2.28 | 0.015263661 |
| *ACAT1* | 2.27 | 0 |
| *SLC25A40* | 2.27 | 0 |
| *SH3GL2* | 2.26 | 0 |
| *CYR61* | 2.26 | 0 |
| *SMAD7* | 2.26 | 0 |
| *C10orf110* | 2.26 | 0 |
| *PDE5A* | 2.26 | 0 |
| *CRIM1* | 2.26 | 0 |
| *MAEL* | 2.26 | 0 |
| *ETV1* | 2.25 | 0 |
| *PMPCB* | 2.25 | 0 |
| *FAM110B* | 2.25 | 0 |
| *MGST3* | 2.25 | 0 |
| *MLLT3* | 2.25 | 0 |
| *VTN* | 2.24 | 0 |
| *FGF9* | 2.24 | 0 |
| *FMO4* | 2.24 | 0 |
| *C2orf88* | 2.23 | 0.015263661 |
| *PROM1* | 2.23 | 0 |
| *STYX* | 2.22 | 0 |
| *ADHFE1* | 2.22 | 0 |
| *BMP4* | 2.22 | 0 |
| *C16orf80* | 2.22 | 0 |
| *TNNI3K* | 2.21 | 0 |
| *ATP1A2* | 2.21 | 0 |
| *KIF13A* | 2.21 | 0 |
| *TSC1* | 2.21 | 0 |
| *DDX1* | 2.20 | 0 |
| *ALDH6A1* | 2.20 | 0 |
| *KLRG1* | 2.20 | 0 |
| *ECHDC1* | 2.20 | 0 |
| *ALS2CR12* | 2.19 | 0 |
| *PDHX* | 2.19 | 0 |
| *MORN4* | 2.19 | 0 |
| *C7orf41* | 2.19 | 0 |
| *UCHL1* | 2.18 | 0 |
| *MOCS2* | 2.18 | 0.015263661 |
| *PER3* | 2.18 | 0 |
| *CCNE2* | 2.18 | 0 |
| *POPDC3* | 2.18 | 0.015263661 |
| *OLFML1* | 2.17 | 0 |
| *SH3BGR* | 2.16 | 0.015263661 |
| *ZNF677* | 2.16 | 0 |
| *MFAP4* | 2.16 | 0 |
| *SERPINE2* | 2.16 | 0 |
| *ENAH* | 2.16 | 0 |
| *GARNL3* | 2.16 | 0 |
| *MOSPD1* | 2.15 | 0 |
| *DHTKD1* | 2.15 | 0 |
| *ATP8A1* | 2.15 | 0 |
| *C7orf64* | 2.14 | 0 |
| *HSPB8* | 2.14 | 0 |
| *APOOL* | 2.14 | 0 |
| *FN1* | 2.14 | 0 |
| *MCCC2* | 2.14 | 0 |
| *CHN1* | 2.14 | 0 |
| *NT5E* | 2.14 | 0 |
| *MYH7B* | 2.14 | 0 |
| *RABGGTB* | 2.14 | 0.015263661 |
| *PKIA* | 2.13 | 0 |
| *EPHA3* | 2.13 | 0 |
| *MYOCD* | 2.13 | 0 |
| *PLCE1* | 2.13 | 0 |
| *C11orf80* | 2.13 | 0 |
| *HIST2H2BF* | 2.13 | 0.015263661 |
| *RFTN2* | 2.13 | 0 |
| *EFCAB2* | 2.13 | 0 |
| *UBE2D1* | 2.13 | 0 |
| *SLC30A1* | 2.13 | 0 |
| *MRPL46* | 2.12 | 0 |
| *SNAP47* | 2.12 | 0 |
| *HLTF* | 2.12 | 0 |
| *PCDH9* | 2.12 | 0 |
| *PRKAB2* | 2.12 | 0 |
| *ADCY5* | 2.12 | 0 |
| *KLRK1* | 2.12 | 0 |
| *NLGN1* | 2.12 | 0 |
| *TMEM38A* | 2.12 | 0 |
| *PRICKLE1* | 2.12 | 0 |
| *CRYZ* | 2.12 | 0.015263661 |
| *C12orf26* | 2.12 | 0 |
| *IRF6* | 2.11 | 0.015263661 |
| *ADH5* | 2.11 | 0 |
| *ATG4C* | 2.11 | 0 |
| *EEF1DP3* | 2.11 | 0 |
| *EGR1* | 2.11 | 0.301941203 |
| *F3* | 2.11 | 0 |
| *DLD* | 2.10 | 0 |
| *ID3* | 2.10 | 0 |
| *PPP2R3A* | 2.10 | 0 |
| *PDE1C* | 2.10 | 0 |
| *ZNF271* | 2.10 | 0 |
| *LIPH* | 2.10 | 0 |
| *KIAA0922* | 2.09 | 0 |
| *ZNF711* | 2.09 | 0 |
| *SLITRK4* | 2.09 | 0 |
| *KLRD1* | 2.09 | 0 |
| *COL1A1* | 2.09 | 0.015263661 |
| *RAB28* | 2.09 | 0 |
| *GADD45G* | 2.09 | 0 |
| *DPM1* | 2.09 | 0 |
| *SNX13* | 2.09 | 0 |
| *NPY6R* | 2.08 | 0.015263661 |
| *ATP5SL* | 2.08 | 0 |
| *LRRC49* | 2.08 | 0 |
| *KLHL13* | 2.08 | 0 |
| *C16orf61* | 2.07 | 0 |
| *XPR1* | 2.07 | 0 |
| *ADI1* | 2.07 | 0 |
| *ATP6V1D* | 2.07 | 0 |
| *YPEL2* | 2.07 | 0 |
| *SOCS2* | 2.07 | 0 |
| *SSPN* | 2.06 | 0 |
| *ASB18* | 2.06 | 0 |
| *PDK1* | 2.06 | 0 |
| *AFF3* | 2.05 | 0 |
| *XRRA1* | 2.05 | 0 |
| *C21orf7* | 2.05 | 0 |
| *EFCAB7* | 2.05 | 0 |
| *GUF1* | 2.05 | 0 |
| *NARS2* | 2.05 | 0 |
| *PGR* | 2.05 | 0 |
| *COPS2* | 2.04 | 0 |
| *EPC2* | 2.04 | 0 |
| *KIF3A* | 2.04 | 0 |
| *NEK10* | 2.04 | 0 |
| *ORC5L* | 2.04 | 0 |
| *FCHO2* | 2.04 | 0 |
| *BMP6* | 2.04 | 0 |
| *SPATS2L* | 2.04 | 0 |
| *MDFIC* | 2.04 | 0 |
| *ABCB7* | 2.04 | 0 |
| *EIF2AK2* | 2.03 | 0 |
| *FIS1* | 2.03 | 0 |
| *SVIL* | 2.03 | 0 |
| *LPL* | 2.03 | 0 |
| *ENC1* | 2.03 | 0 |
| *CROT* | 2.03 | 0 |
| *SC4MOL* | 2.03 | 0.015263661 |
| *GLIPR1* | 2.03 | 0.015263661 |
| *ACTG2* | 2.02 | 0 |
| *GFPT1* | 2.02 | 0 |
| *SERPINI1* | 2.02 | 0 |
| *CLYBL* | 2.02 | 0 |
| *ACADSB* | 2.02 | 0.015263661 |
| *CPEB3* | 2.02 | 0 |
| *ZNRD1* | 2.02 | 0 |
| *AEBP1* | 2.02 | 0 |
| *GALM* | 2.02 | 0 |
| *C6orf203* | 2.02 | 0.033807449 |
| *FBXO40* | 2.02 | 0 |
| *SPIN1* | 2.02 | 0 |
| *MTERFD3* | 2.01 | 0 |
| *SAMD9* | 2.01 | 0 |
| *GPX8* | 2.01 | 0 |
| *C1orf51* | 2.01 | 0 |
| *FH* | 2.01 | 0 |
| *C18orf55* | 2.01 | 0 |
| *MTHFD2L* | 2.01 | 0 |
| *CASQ2* | 2.01 | 0 |
| *PDHB* | 2.01 | 0 |
| *SYTL5* | 2.00 | 0 |
| *GEM* | 2.00 | 0 |
| *C8orf37* | 2.00 | 0 |
| *NT5C3* | 2.00 | 0 |
| *DCLK2* | 2.00 | 0 |
| *ANKRD29* | 2.00 | 0 |
| *ACAD8* | 2.00 | 0 |

Table S2b: List of 108 downregulated genes in the explant/failed heart.

|  | | |
| --- | --- | --- |
| *Gene ID* | Fold Change | q-value(%) |
| *PLA2G2A* | 0.02 | 0 |
| *CA1* | 0.06 | 0 |
| *IL1RL1* | 0.07 | 0 |
| *UGT2B4* | 0.10 | 0 |
| *GSTT1* | 0.11 | 0 |
| *HBB* | 0.11 | 0 |
| *SERPINA3* | 0.13 | 0 |
| *MMP8* | 0.14 | 0 |
| *STC1* | 0.14 | 0 |
| *C3* | 0.14 | 0 |
| *F5* | 0.15 | 0 |
| *CYP4B1* | 0.16 | 0 |
| *PPP1R1A* | 0.17 | 0 |
| *ADAMTS9* | 0.18 | 0 |
| *ALAS2* | 0.18 | 0 |
| *CORIN* | 0.20 | 0 |
| *ADAMTS5* | 0.20 | 0 |
| *AQP4* | 0.21 | 0 |
| *STEAP4* | 0.22 | 0 |
| *FABP2* | 0.23 | 0 |
| *MGST1* | 0.24 | 0 |
| *SLCO4A1* | 0.24 | 0 |
| *TC2N* | 0.24 | 0 |
| *PDK4* | 0.25 | 0 |
| *FCN3* | 0.26 | 0 |
| *SGPP2* | 0.26 | 0 |
| *S100A9* | 0.27 | 0 |
| *EDNRB* | 0.28 | 0 |
| *IL18R1* | 0.28 | 0 |
| *GFPT2* | 0.29 | 0 |
| *KCNIP2* | 0.29 | 0 |
| *RARRES1* | 0.30 | 0 |
| *ERAF* | 0.32 | 0 |
| *ANGPTL4* | 0.32 | 0 |
| *GALNTL2* | 0.32 | 0 |
| *AQP3* | 0.33 | 0 |
| *LOC284757* | 0.33 | 0.102880443 |
| *HVCN1* | 0.34 | 0 |
| *MYC* | 0.34 | 0 |
| *OSMR* | 0.35 | 0 |
| *CEACAM8* | 0.36 | 0 |
| *FLJ23834* | 0.36 | 0 |
| *CHL1* | 0.36 | 0 |
| *C4B* | 0.36 | 0 |
| *IGFBP4* | 0.36 | 0 |
| *CATSPERB* | 0.37 | 0 |
| *CP* | 0.37 | 0 |
| *MYH6* | 0.37 | 0 |
| *FAM107A* | 0.37 | 0 |
| *DARC* | 0.37 | 0 |
| *HBD* | 0.38 | 0 |
| *AKR1C4* | 0.38 | 0 |
| *C1orf105* | 0.39 | 0 |
| *DEFA4* | 0.39 | 0 |
| *SYN2* | 0.39 | 0 |
| *C1R* | 0.40 | 0 |
| *ETS2* | 0.40 | 0 |
| *LTF* | 0.41 | 0 |
| *IRAK3* | 0.41 | 0 |
| *C1RL* | 0.41 | 0 |
| *C1QTNF1* | 0.42 | 0 |
| *AQP1* | 0.43 | 0 |
| *IL1R2* | 0.43 | 0 |
| *CLEC12A* | 0.43 | 0 |
| *ICAM2* | 0.43 | 0 |
| *CAMK1D* | 0.44 | 0 |
| *IL6* | 0.44 | 0 |
| *AOX1* | 0.44 | 0 |
| *STON1-GTF2A1L* | 0.44 | 0 |
| *CHRNA7* | 0.45 | 0.015263661 |
| *C10orf10* | 0.45 | 0 |
| *CD38* | 0.45 | 0 |
| *PAPSS2* | 0.45 | 0 |
| *CHST9* | 0.45 | 0.102880443 |
| *RHOU* | 0.45 | 0 |
| *LRRC7* | 0.46 | 0 |
| *MMP9* | 0.46 | 0 |
| *REPS2* | 0.46 | 0 |
| *TCN1* | 0.46 | 0 |
| *STAT3* | 0.46 | 0 |
| *EXPH5* | 0.46 | 0 |
| *LCN2* | 0.46 | 0 |
| *NNMT* | 0.46 | 0 |
| *IL18RAP* | 0.46 | 0 |
| *VWF* | 0.47 | 0 |
| *OLFM1* | 0.47 | 0 |
| *CEBPD* | 0.47 | 0 |
| *GPC5* | 0.47 | 0 |
| *SLCO2A1* | 0.47 | 0.015263661 |
| *CR1* | 0.47 | 0 |
| *RNF122* | 0.47 | 0 |
| *CD177* | 0.48 | 0 |
| *TOX* | 0.48 | 0 |
| *SERPING1* | 0.48 | 0 |
| *C13orf33* | 0.48 | 0 |
| *RDH10* | 0.48 | 0 |
| *THBD* | 0.48 | 0 |
| *MPO* | 0.49 | 0 |
| *TMEM176A* | 0.49 | 0 |
| *F11R* | 0.49 | 0 |
| *FAM89A* | 0.49 | 0 |
| *C20orf54* | 0.49 | 0 |
| *TPO* | 0.49 | 0 |
| *NP* | 0.49 | 0.033807449 |
| *HYAL2* | 0.49 | 0 |
| *LDB2* | 0.50 | 0 |
| *SCARA5* | 0.50 | 0 |
